# CAR T Cell Therapy: A Versatile Living Drug

**DOI:** 10.3390/ijms24076300

**Published:** 2023-03-27

**Authors:** Rodrigo C. De Marco, Hector J. Monzo, Päivi M. Ojala

**Affiliations:** 1 Faculdade de Engenharia da Universidade do Porto (FEUP), 4200-465 Porto, Portugal; 2 Instituto de Ciências Biomédicas Abel Salazar (ICBAS), Universidade do Porto, 4050-313 Porto, Portugal; 3 Translational Cancer Medicine Research Program, Faculty of Medicine, University of Helsinki, 00014 Helsinki, Finland

**Keywords:** chimeric antigen receptor, adoptive T cell transfer, immunotherapy

## Abstract

After seeing a dramatic increase in the development and use of immunotherapy and precision medicine over the past few decades, oncological care now embraces the start of the adoptive cell therapy (ACT) era. This impulse towards a new treatment paradigm has been led by chimeric antigen receptor (CAR) T cells, the only type of ACT medicinal product to be commercialized so far. Brought about by an ever-growing understanding of cellular engineering, CAR T cells are T lymphocytes genetically modified with an appropriate DNA construct, which endows them with expression of a CAR, a fusion protein between a ligand-specific recognition domain, often an antibody-like structure, and the activating signaling domain of the T cell receptor. Through this genetic enhancement, CAR T cells are engineered from a cancer patient’s own lymphocytes to better target and kill their cancer cells, and the current amassed data on clinical outcomes point to a stream of bright developments in the near future. Herein, from concept design and present-day manufacturing techniques to pressing hurdles and bright discoveries around the corner, we review and thoroughly describe the state of the art in CAR T cell therapy.

## 1. Introduction

Cancer, also referred to as malignant tumor, develops from neoplastic tissues and can generally be defined as a group of transformed cells which present abnormal, uncontrolled growth and are capable of invading neighboring tissues and/or metastasizing to regional lymph nodes and/or distant organs [1]. The term cancer actually encompasses a wide variety of diseases, distinguishable by organ or tissue of origin and cell type and morphology [2]. It is currently the largest contributor to disability-adjusted life years and the second-highest cause of worldwide mortality [3]. In addition, it is expected to rise to the leading cause of death in the next decades, surpassing ischemic heart diseases [3].

The fruit of frantic and unruly proliferation, many of the cancerous tumor cells become riddled with a wide number of accumulated mutations that provide them with mechanisms that exacerbate growth, prevent apoptosis and, among other hallmarks, help to evade immune surveillance [4,5]. An example of the latter is the frequent downregulation of major histocompatibility complex (MHC) molecule expression in cancer cells and alterations to their antigen processing and loading, which result in hindered oncoantigen (or neoantigen) presentation and subsequent reduced visibility to the immune system, leading to an atrophied response [5,6]. On top of this, the increased mutagenesis that occurs due to the intrinsic genomic instability of cancer cells contributes to the creation of distinct subclonal heterogeneous lineages with different capabilities, such as invasion, increased proliferation, drug-resistance, and oncoantigen/neoantigen presentation [4,7,8].

Throughout modern history, many different strategies have arisen for treating these malignancies. Surgery, chemo-, and radiotherapy have had undeniable success in reducing cancer-related mortality and improving cure perspectives, with chemotherapy being a go-to treatment for basically all hematological cancers and most cancers in general. However, beyond unavoidable case-to-case variability and subsequent suboptimal efficacy, these so-called conventional therapies are associated with severe side effects, caused by the collateral damaging of healthy tissues, and sometimes long recovery [9,10].

Thus, development and employment of new treatment concepts have been initiated to further improve overall survival and reduce cancer burden, while inducing lower systemic toxicity. One of them was immunotherapy, which uses antibodies, cytokines, and immune cells to modulate the host immune response to cancer [9,11,12].

Recently, and building upon the advent of targeted and immune therapies and their advantages, adoptive cell therapies (ACTs) have gained considerable traction in the field of oncology [13]. These strategies are based on the infusion of lymphocytes, usually autologous T cells, to fight disease in patients. By selecting or modifying the lymphocytes’ specificity towards a target antigen, they are expanded and injected back into the patient, where they exert their cytotoxic activity and help to mount a sustained immune response against it [14].

In chimeric antigen receptor (CAR) T cell therapy, autologous T cells are isolated from the patient’s peripheral blood, endowed with enhanced specificity and killing efficacy towards the patient’s cancer cells, and then reinjected into the host, where they will aid in tumor clearance. This is achieved through genetic modification of the T cells so that they express the CAR, a receptor engineered to recognize a given antigen of the patient’s cancer cells and activate the CAR T cells’ expansion and cytotoxic potential upon recognition [15,16].

Though the basic structure of the CAR is relatively well-defined, a chimeric receptor’s specific design can be reshaped anew with ease, as it is composed of several regions of different proteins stitched together into one fusion protein [17,18], or “chimera”. Part of the CAR’s tailoring involves deciding on an appropriate and effective recognition domain, so that the modified T cells target a desired predefined antigen, like a tumor-associated antigen (TAA), for example. This represents a great advantage as, opposed to regular T cells that rely on antigen presentation through MHC expression, CAR T cells are unbound by these constraints and are instead capable of recognizing all expressed surface antigens, even if they are not MHC-presented [14]. Thus, even the more “invisible” cells that downregulate antigen presentation are detected, resulting in a greater pool of targets and therefore enhanced overall killing efficacy.

## 2. How Do CAR T Cells Kill?

The way in which CAR T cells activate and kill is usually thought to be quite similar to normal T cell receptor (TCR) signaling. Yet newly found discrepancies keep being brought up by novel studies [19]. For example, the protein pattern in CAR T cell immune synapses is more disorganized than that of physiological T cells, accelerating lytic action and affecting effector–target dissociation kinetics [20].

**Figure 1:**
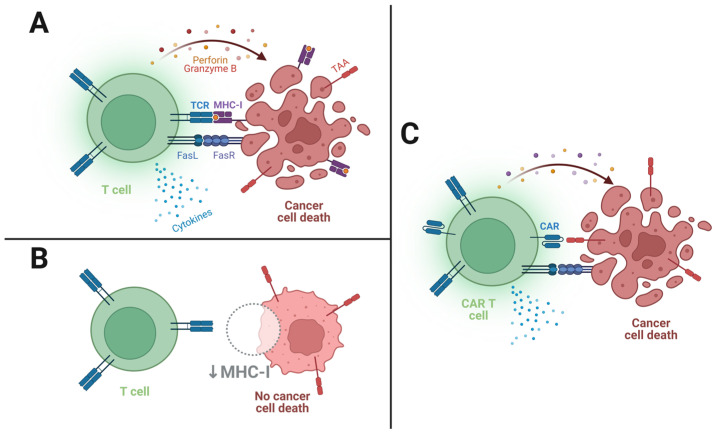
Schematics of T cell and CAR T cell killing mechanisms. (**A**) TCR recognition of a cancer cell’s TAA epitope presented on MHC-I induces killing of the target cell; (**B**) Downregulation of MHC-I by the cancer cell prevents T cell activation and subsequent target cell killing; (**C**) CAR recognition of the surface-expressed TAA triggers killing mechanisms despite MHC-I downregulation.

Briefly, upon initial ligand recognition, a non-classical immune synapse between the effector and target cell is formed, a small and tight space where multiple receptor–ligand associations occur in proximity. This synaptic receptor-clustering event leads to activation, a complex cellular program that includes clonal expansion and target cell killing. Cytotoxic effects can be achieved through two important pathways (see Figure 1 below): membrane expression of TNF ligands, which induce apoptosis when recognized by their receptor (such as FasL/FasR), called “slow-acting killing mechanisms”; and the “fast-acting” exocytosis of perforin and granzyme granules, which open pores in the target cell’s plasma membrane and force apoptosis through caspase-dependent and caspase-independent pathways [21]. Moreover, activated CAR T cells secrete cytokines, such as IL-2, IL-6, and IFN-γ, that recruit and potentiate the action of other immune cells, like NK cells, macrophages, and other T cells, orchestrating a more robust tumor-suppressive environment [22].

## 3. Currently Available CAR T Cell Therapies

Within the different types of ACTs, CAR T cell therapy stands as the only one that has received commercialization approval from either the FDA (Food and Drug Administration) or EMA (European Medicines Agency), with six different products authorized for use in the USA and in Europe, used for the treatment of a total of seven B cell malignancies (see Table 1). Most approved products use anti-CD19 CAR T cells, engineered to target and kill cells that express the pan-B cell marker, and the results have proven to be quite impressive. The outstanding success associated with this target therapy can mostly be explained by the following two reasons:

1. CD19 expression is both quite limited to, and ubiquitous in, B cells [23]; therefore, its targeting avoids toxicity to other tissues while assuring the targeting of malignant B cells;
2. As the anti-CD19 CAR T cells target all B cells in the patient’s body, the therapies will frequently cause B cell aplasia and consequent hypogammaglobulinemia (low serum antibody levels) [24]. Fortunately, although debilitating as they may be, these conditions are compatible with resuming a relatively normal life, especially with the use of immunoglobulin infusions [25].

Antigens like CD20 and CD22, which have had some success in immunotherapies, are also under investigation for possible CAR T development [26], along with HER2, IL13Rα2, and others.

It is also worth noting that hematological malignancies have been far better targets for CAR T cell therapies than solid tumors, partly due to the massive differences in tumor stroma permissiveness. Briefly, in blood malignancies, CAR T cells are not required to penetrate through dense layers of extracellular matrix (ECM) to get to the tumor cells, so access to target cells is considerably easier.

Although, as of now, commercially available CAR T cell therapies are few, and therefore products are well-defined formulas, one can envision that, in the future, they will be selected or even custom made for each patient, considering the specific characteristics of their particular illness, under the concepts of precision medicine and personalized therapy [27]. Heading into another direction, though, research attempts towards developing off-the-shelf, standardized allogeneic products are also grabbing attention. However, concerns over graft-versus-host disease (GVHD) and low persistence seem to be, at the moment, holding back any major breakthroughs [28].

### Current Status

A major reason as to why adoptive CAR T cell transfer has been drawing so much attention lately is the astonishing results that it has achieved.

In one of the first phase 1 CAR T cell clinical trials, a patient who was suffering from refractory B cell chronic lymphocytic leukemia for over 10 years was submitted to anti-CD19 CAR T cell infusion. Prior to CAR T cell treatment, the patient had undergone several treatments of chemo- and immunotherapy. At first, there was a response, but after a couple of relapses and treatment cycles, therapeutic efficacy dropped almost completely. However, at 23 days post-CAR T cell infusion, no leukemic cells were detected in the bone marrow and, five days later, previously present adenopathy was not reported upon physical examination. Moreover, the patient showed sustained remission in the follow-ups leading up to the completion of the study, evaluated through morphological, karyotypical, and flow-cytometric analyses [29].

Following promising in vitro and in vivo preclinical results, Dr. Sadelain (a pioneer in T cell engineering and CAR T cells), Dr. Brentjens, and colleagues published, in 2013, the first clinical trial results of CAR T cell therapy in B cell acute lymphoblastic leukemia (B-ALL). They reported tumor eradication, complete remission, and also negative minimal residual disease (MRD^−^) for the whole patient cohort [30].

In a 2014 clinical trial [31], after an initial success the year before [32], 30 patients suffering from B-ALL (including both children and adults) were treated with CTL019, a CAR T cell therapy product that was later named Kymriah (tisangenlecleucel) and that eventually became the first approved CAR T cell product in 2017. All patients had either relapsed or refractory (r/r) disease, meaning their cancer recurred or did not respond to standard therapies to begin with. Of the 30 patients, considered to be incurable, 27 (90%) achieved complete remission and 22 showed MRD^−^ one month after infusion. Six months post-infusion, the overall survival rate was of 78% and the event-free survival rate reached 67%. By the end of the two-year-long cohort, 19 patients were reported to still be in remission, most with no further therapy [31].

In a later clinical trial, sponsored by the National Cancer Institute [33], fifteen patients with advanced r/r CD19^+^ B cell malignancies were treated with an anti-CD19 CAR T cell therapy. Of these patients, nine were stricken with diffuse large B cell lymphoma, two with indolent lymphoma, and four with chronic lymphocytic leukemia. Following CAR T treatment, eight complete remissions, four partial remissions, and one stable disease were achieved.

Kymriah (tisangenlecleucel), the first CAR T product to be granted marketing approval, is a product consisting of autologous T cells transduced with a CAR composed of an anti-CD19 scFv, a CD8α hinge and transmembrane domain, CD28 costimulatory and CD3ζ signaling domains (19.28.z). Clinical data highlight that Kymriah usually underwent rapid expansion after infusion and, after two years, was still detectable in r/r B-ALL patients’ blood and bone marrow. Its expansion correlated with initial tumor burden, and treatment for therapy-induced cytokine release syndrome (CRS) did not seem to reduce its function. Overall response rates and MRD^−^ patient proportion reach as high as 80% three months after treatment, with 60% reaching complete remission, which was considered a significant increase for r/r B-ALL patients [34].

Research points out that some CAR T cell therapies aimed at treating T cell acute lymphoblastic leukemia might be hindered by T cell fratricide. However, protein expression-blocking strategies can decrease CAR T/CAR T fratricide, as stated by Png et al. [35].

Many more trials and treatment data keep vouching for the undeniable potential of CAR T cell therapy for various clinical conditions. However, toxicity and inconsistent persistence are issues for which management strategies are still being explored.

Below, in Table 2, a short summary of recent clinical results of currently available CAR T cell therapies and some selected clinical trials can be found. It highlights the astounding success of some therapies, namely the ones on the market, which have achieved high response rates, helped to halt disease progression, and improved overall survival. Patients burdened with advanced malignancies used to face dismal prognoses, with as low as 6 months of median overall survival [36]. The table also highlights some of the pitfalls of CAR T cell therapy, especially in the solid tumor setting, such as antigen escape and high-grade adverse effects.

## 4. Structure of the CAR

The CAR modular composition provides CAR T cell therapies with an unparalleled tunability of effector cell properties (such as affinity, persistence, and potency), and, consequently, great versatility in medical applications. In fact, through simple plasmid editing and cloning, it is quite straightforward to insert, alter, or delete domain-encoding sequences and build a custom CAR construct.

CARs are membrane-bound signaling receptors composed of ligand-binding and spacer ectodomains, a transmembrane domain, and one or more cytoplasmic domains. The outermost region consists of the ligand-binding domain (henceforth abbreviated as LBD), which is responsible for antigen recognition. The cytoplasmic region, comprised by the endodomains, is linked to the ectodomains by the transmembrane domain, which keeps the receptor membrane bound. When the receptor binds to its target antigen, conformational changes take place throughout the whole protein. In the endodomains, these conformational changes alter the exposure and reactivity of certain amino acids, leading to the catalysis of several post-translational modifications (PTMs) that commence intricate intracellular signaling cascades, culminating in T cell activation.

### 4.1. Ligand-Binding Domain

LBDs are responsible for binding and recognition of a specific antigen. Some important characteristics of this region are its steric hindrance, avidity, and affinity towards the target antigen, as they affect binding probability and strength. Given its modular structure, it is possible to select or create a specific CAR construct to the detriment of similar ones to ensure a desired ligand-binding characteristic, such as a higher or lower affinity. This becomes increasingly important as the scientific community continues to realize that, depending on the application and CAR construct, modulation and appropriate tuning of these properties can improve therapeutic efficacies and safety. In fact, studies have shown that lower-affinity CARs might reduce CAR T cell fratricide induced by spontaneous trogocytosis, improving CAR T cell persistence and maintaining high efficacy [46].

Other LBD properties can modulate the CAR T cell response, such as frequency and intensity of stabilizing interdomain interactions, propensity for synapse formation, and receptor dimerization, as well as synapse density and pattern. This is due to the fact that these influence the probability of self-activation or activation under rightful target recognition [21]. Studying and understanding these mechanisms will surely shine some light on ways of fine-tuning CARs for more appropriate treatment personalization.

#### 4.1.1. scFv

Composed of an antibody’s variable fragment heavy (V_H_) and light (V_L_) chains connected by a linker peptide, the most common type of LBD used in CARs is the single-chain variable fragment (scFv). The base antibody may be of animal origin, humanized, or of human origin, which are each associated with different levels of effectiveness and elicited adverse immune response, but also manufacturing costs. Medical therapies preferably rely on the use of xeno-free products to avoid adverse immune reactions, meaning that scFvs should originate from human antibodies, or humanized ones at least, while in basic research, more inexpensive xenogeneic products may be the preferred option. Still, many of the currently available CAR T cell therapies possess xenogeneic scFvs [47].

It is important to mention the role of the linker peptide in CAR function, as proximity and mobility of scFv chains are crucial to proper antigen recognition and CAR homodimerization, influencing subsequent activation and cell-killing effects. Linkers usually follow the (GGGGS)_n_ structure, meaning the amino acid sequence between V_H_ and V_L_ consists of *n* (1 to 4) repetitions of the Gly-Gly-Gly-Gly-Ser peptide. Dr. Singh’s recent findings point to a heightened effector capability of anti-CD22 CAR T cells possessing smaller linkers due to increased homodimerization propensity [48].

#### 4.1.2. Other LBDs

Although scFvs are the most widely used recognition domains in CAR T cells, and currently the only ones in commercially available CAR T cell products, alternatives have been subject to experimentation and their possible use in clinical settings draws progressively more of the scientific community’s attention. Alternative LBDs include:

- common ligand receptors—e.g., NKG2D [49,50];
- ligands, in a reverse approach, when the target molecule has a receptor function—e.g., IL-11 [51];
- nanobodies, which are composed of a single antibody variable domain (VHH) [52];
- antibody mimetics [53].

### 4.2. Spacer Domain

The spacer domain is, in its essence, a hinge, a region of the extracellular portion of the CAR that plays an important role in determining its outer length and reach, placing the scFv out of the cell’s glycocalyx and allowing for greater LBD mobility. Thus, the spacer domain dictates where binding occurs beyond the membrane, and how often (or probable) it is to occur. Regarding the binding location within the synaptic cleft, studies have come to show that the distance at which ligand recognition takes place affects activation, since the length separating the two plasma membranes is crucial to optimal synapse function [54]. Indeed, even though CAR T cells form non-classical immune synapses, their cytolytic granule secretion and segregation kinetics are still key factors in their signaling and cytotoxicity [55]. It is therefore logical that, for each LBD–ligand pair, there exists a spacer which determines ideal distancing. In the case of mobility, if an antigen is sterically hindered, a CAR with a spacer that imparts more mobility to the LBD should prove advantageous over a CAR with a stiffer spacer region [56]. Some spacers used in clinical practice include CD28 and CD8α hinges, which can confer different levels of antigen threshold in relation to activation and activation-induced cell death (AICD) [55].

### 4.3. Transmembrane Domain

The function of the transmembrane domain consists of anchoring the CAR to the cellular membrane, linking the LBD to the endodomains, and keeping the first in contact with the external environment while the latter remain embedded in the cytosol. As per the findings of Guedan et al., constructs differing only in this domain can lead to different levels of T cell activation upon binding, also entailing an effect on signal strength activation [57].

Often, the transmembrane domains utilized coincide with either spacer or endodomain regions of the construct. As such, CD8, CD28, or ICOS transmembrane regions are commonly seen in CARs.

### 4.4. Endodomains

The cytoplasmic domains of the CAR have the crucial role of initiating intracellular signaling cascades triggered by ligand recognition. Findings suggest that the extracellular domains induce endodomain conformational changes, and then chemically reactive groups in select amino acids of the latter become more or less accessible to the local environment. This, in turn, allows for processes such as PTMs and receptor dimerization to occur, culminating in activated catalytic function of these domains. These activated enzymatic regions then act upon their designated intracellular substrates (usually other signaling proteins, namely other enzymes), starting a succession of enzyme–substrate interactions that eventually alter gene expression and modulate defined cellular programs, influencing the overall cellular status [58,59]. In competent CAR T cells, upon binding to the target antigen, synapse formation, CAR dimerization, and enzymatic activation take place, leading to the initiation of the signaling cascades.

#### 4.4.1. CD3ζ Signaling Domain

The CD3ζ-chain of the TCR is a domain with inducible tyrosine kinase function. It contains three immunoreceptor tyrosine activation motifs (ITAMs), which can produce an activating signal strong enough on its own to be used in the absence of the other TCR domains. The ζ-chain is responsible for the specificity signal in T cells, or signal 1, which is required to kick-start T cell activation and induce IL-2 production, widely known to be a crucial promoter of T lymphocyte expansion [14,21,26]. As such, the CD3ζ-chain is considered a mandatory endodomain to be included in all CAR constructs. Signals 2 and 3, produced by costimulatory molecules and cytokines, respectively, modulate the output of signal 1, amplifying its effects.

In normal T cell signaling, when the TCR recognizes its cognate antigen, several protein tyrosine kinases bind and phosphorylate the CD3 ITAMs (or help to phosphorylate/prevent phosphatase action). Some of the most crucial of these enzymes include LCK, FYN, and ZAP-70, which initiate the canonical TCR signaling pathways. Distal effects of these complex cascades contemplate the activation of the NFAT, NF-κB, and mTOR pathways, which stimulate growth and survival, cell cycle progression and cellular division, and cytokine secretion [60]. Although some discrepancies may exist, CAR T cell activation signaling is believed to follow the same basic pathways as the ones described for regular T cell signaling.

#### 4.4.2. Costimulatory Domains

After some early experimentation, it soon became clear that genetically engineered lymphocytes expressing CARs with only the CD3ζ-chain of the TCR were able to activate and produce a cytotoxic response, but not to maintain it and persist. Much more rapidly than normal T cells, first-generation CAR T cells developed anergy (irresponsiveness to expansion stimulation) and suffered from intense AICD. It was determined that the lymphocytes were missing an important piece of the signaling puzzle: costimulatory domains (CsDs). Regular T cells express proteins which are responsible for modulating T cell activation—the costimulatory molecules; they bind to ligands expressed by various cells, namely antigen-presenting cells, and, when activated, these costimulatory molecules produce signal 2 of T cell signaling, boosting the effects of signal 1, effectively prolonging survival, sustaining proliferation, and potentiating the release of cytokines and cytolytic granules [26,60].

Many T cell costimulatory molecules are known, namely CD28, 4-1BB, OX-40, ICOS, CD27, and CD40L [61]; however, as of now, in clinical settings, only CD28 and 4-1BB CsDs are used in commercially available second-generation CAR T cell products. Since only these CsDs are in use, knowledge of them has stockpiled high in comparison to the rest. Research and treatment data have elucidated that the two CsDs have different effects on the lymphocytes:

- CD28 heightens the signaling speed and intensity and cytokine production, induces effector memory differentiation on the activated CAR T cells, and reduces effects of inhibitory molecules and Tregs, increasing the CAR T cells’ cytolytic and inflammatory potential; however, it imparts a tendency for fast exhaustion, and thus lower persistence, than 4-1BB-containing second-generation products [55]. The more “explosive” behavior of CD28 CAR T cells seems to also bring along a downside of greater chances for and intensity of CRS and neurological toxicity [62,63].
- 4-1BB, on the other hand, induces greater persistence and a central memory phenotype without such exacerbated toxicity, at the cost of slower and less intense signaling and cytotoxic activity, offering instead a more sustained anti-tumor activity [55,63].

Either CsDs can be more or less adequate depending on disease characteristics and patient condition, among other factors, and more research is required to assert clearly which are the preferrable treatments for each clinical situation.

It is also worth noting that the order of CsDs within the CAR construct affects function: Generally speaking, CsD positioning between the transmembrane domain and the activating domain, leaving the ζ-tail “hanging” in the cytosol, seems to impart superior effector capacity [55].

### 4.5. CAR T Generations According to Structure

The first CAR constructs generated and proven to work had an intracellular region composed of only the CD3ζ endodomain—first-generation CARs, whose creation was initially described in the seminal work of Dr. Eshaar, back in the 1990s [64]. However, it has since then become clear that the ζ-chain alone is insufficient to elicit a complete T cell response and avoid exhaustion and subsequent AICD. Thus, second- and third-generation CARs arose that have one or two costimulatory endodomains, respectively, added to the construct for improved lymphocytic action and persistence [55,65]. These CsDs empower the effects of the CD3ζ activating signal by initiating parallel intracellular signaling cascades that enhance proliferation, cytotoxic action, cytokine release, cell survival, and memory formation [66].

**Figure 2:**
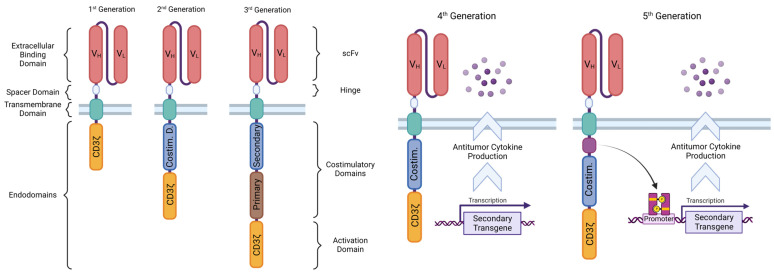
Chimeric antigen receptor designs, ordered by their corresponding conceptual generation.

Nowadays, experimental fourth- and fifth-generation CARs are being tested, which can incorporate constitutive or inducible transgenic signaling, respectively, such as that of cytokines (**T** cells **R**edirected for Antigen-**U**nrestricted **C**ytokine-Initiated **K**illing—TRUCKs) and chemokines (self-driving CARs). Special CAR constructs are also under research that make use of new types of LBDs or implement more complex targeting strategies, such as dual targeting and logic-gated signaling [19,26]. Of note is that unanimous nomenclature is still to be agreed upon for some of these novel and exciting concepts. As such, different names can be seen attributed to the same CAR design throughout the literature (or vice versa). Figure 2 represents the CAR designs throughout their generations.

## 5. CAR T Cell Processing

### 5.1. Manufacturing

The manufacturing of CAR T cells is a multi-step, one-to-two-week-long ex vivo process which deeply affects functionality, and thus influences the outcomes of both preclinical results and therapy.

Briefly, the general manufacturing process is initiated by the collection and isolation of peripheral blood mononuclear cells (PBMCs). This can be performed by collecting the patient’s blood and separating it into different components (e.g., using density-gradient centrifugation with Ficoll-Paque) or directly through leukapheresis, a type of apheresis that automatically isolates leukocytes. Next, CD4^+^ and/or CD8^+^ T cells (depending on the desired end product) are either enriched from the initial PBMC sample and subsequently activated, or vice versa. Ex vivo activation relies on exposure to plate-, nanomatrix-, or beads-bound activating anti-CD3 and anti-CD28 antibodies [67,68]. The following step is the genetic modification of the isolated T cells, which can be done through either viral or non-viral approaches that drive the CAR construct into the lymphocytes. Usually, the construct is encoded by DNA and integrates into the T cell genome, imparting permanent expression. RNA-based constructs are also sometimes used and permit transient expression, which can help to improve toxicity profiles. Viral approaches stand as the most used for CAR T cell production, and normally use lentiviral or γ-retroviral vectors, which are expensive but present great transduction efficiency. On the other hand, non-viral vectors like transposon-derived plasmids trade some efficiency for reduced costs and are therefore slowly growing in popularity, especially when it comes to research into CAR T cell mass production [68,69]. Further CAR T cell expansion may then be performed to meet clinical-grade criteria for administration (besides required good manufacturing practice conditions) or simply to increase the number of effector cells available for research purposes [68].

**Figure 3:**
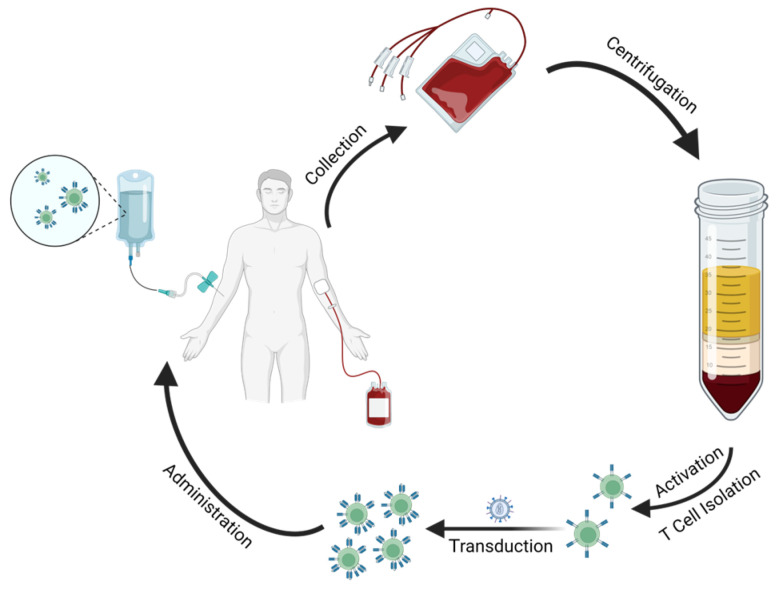
Simplified visualization of the steps involved in an example manufacturing process for CAR T cells.

Figure 3 depicts an example of CAR T cell product manufacturing, consisting of patient blood collection, PBMC isolation through density-gradient centrifugation, T cell isolation and activation, followed by transduction with a CAR viral vector and final ex vivo expansion, culminating in end product administration.

### 5.2. Administration

Upon treatment, around 10^8^ viable CAR T cells are infused intravenously into patients in a certified healthcare facility (rough estimate—information retrieved from Kymriah’s, Breyanzi’s, and Yescarta’s FDA package inserts). A few days before infusion, however, patients go through lymphodepleting conditioning, a type of chemotherapy that targets the patient’s immune cells—usually by fludarabine and cyclophosphamide. This procedure has been shown to allow for greater CAR T cell expansion and persistence post-infusion, and greater antitumoral efficacy [70,71]. It is hypothesized that lymphodepleting regimens are beneficial through multiple ways: elimination of immunosuppressive cells, such as Tregs and myeloid-derived suppressor cells (MDSCs); reduction of anti-CAR response by host lymphocytes; depletion of homeostatic cytokine reservoirs that induce increased immunoproliferative signaling, resulting in peaks of immunostimulatory cytokines and effector cell function when these are infused; and modulation of tumor cells and microenvironment [47,71].

As available CAR T cell therapies are autologous, patients need to wait and survive through their long and costly manufacturing process. This is especially critical as patients eligible for CAR T cell therapy suffer from advanced malignancies that quickly exact a high toll on their health status. Thus, bridging therapy becomes an important part of patients’ health, keeping disease progression at bay between the failures of previous treatment attempts and CAR T cell therapy. Bridging therapy regimens consist of immunotherapy, and/or chemo- or radiotherapy, often including targeted strategies [71].

## 6. Current Challenges

CAR T cell therapies have shown outstanding results, u-turning dismal outlooks and saving many patients from aggressive malignancies. Even so, their pitfalls are continuously being brought to light by ever-growing accumulated clinical experience. Challenges that, as of now, plague approved treatments and stand in the way of the development of novel, improved therapies include low CAR T cell persistence, antigen escape events, insufficient tumor-killing efficacy (especially in the clearance of solid tumors), and high toxicity profiles, namely cases of severe CRS and neurotoxic side effects. Also of note are the high costs of current available products and the already mentioned prolonged manufacturing period [72].

### 6.1. Efficacy and Persistence

It has been hypothesized that low levels of basal, unstimulated oligomerization of CARs induce a state of tonic signaling in CAR T cells that makes them more susceptible to succumb to anergy and exhaustion, leading to lower reactivity and persistence [19]. Furthermore, even if remission rates have been truly baffling, considering the dire clinical status of patients, many tumors still resist therapy or end up relapsing sometime after, mostly due to insufficient persistence or unrestrained growth of selectively favored subclone lineages, like antigen-negative cell populations. Meanwhile, attempts to apply CAR T cells to solid tumors have fallen short of expectations. These challenges have been the subject of great research efforts from the scientific community in the last few years, as overcoming them would not only improve the applicability of these promising therapies, but their overall results as well.

#### 6.1.1. Tumor-Antigen Escape

Benefited by an increase in selective pressure, refractory cancer subclones often overtake tumors after the administration of a targeted therapy. In large B cell lymphoma patients [73], anti-CD19 CAR T cells effectively eliminate most of the CD19^+^ cancer cells; however, CD19^−^ cancer cells escape the targeted therapy, and can then proliferate at will and perpetuate the cancer [74]. 

CD19^−^ escape is, to this date, one of the main causes of relapse in hematological malignancies after CAR T cell treatment, and strategies that allow the overcoming of this hurdle would significantly increase disease- and progression-free survival rates [26].

#### 6.1.2. Solid Tumors

Thus far, the remarkable success of adoptive CAR T cell transfer has had a revolutionary impact in the treatment of hematological cancers. Alas, despite these efforts, this is not the case (yet) for solid ones [75,76].

Multiple reasons, inherent in the nature of solid tumors, help to justify the impaired reach and effectiveness of CAR T cells in this context:

1. First and foremost, CAR T cells are infused into the blood, and must then traffic to the region where the tumor is located, a process which is dependent on chemokine attraction signals and is therefore variable from tumor to tumor [75].
2. After reaching the tumor site, the lymphocytes must penetrate through the layers of ECM, frequently thickened and stiffened by intense collagen and heparan sulphate proteoglycan deposition carried out by tumor-associated fibroblasts. To make matters worse, T cells do not produce significant amounts of ECM-degrading enzymes, meaning they tend to move less through areas with a denser matrix. Hence, this barrier hinders deeply the accessibility of CAR T cells to their target cells [77].
3. Atop this, the microenvironment of solid tumors is often oxidative, hypoxic, acidic, and nutrient-starved, and consists of high amounts of immunosuppressive elements, be they cytokines and other soluble factors, cells (e.g., Tregs, tumor-associated macrophages (TAMs) and MDSCs), or even the tumor cells themselves, through the expression of ligands like CTLA-4 and PD-L1. This immunologically deleterious “cold tumor” environment spurs the development of anergic and apoptotic states in the CAR T cells [78].
4. Unlike B cell malignancies, it has proven challenging to find TAAs that are specifically yet uniformly expressed in the tumor at high levels. TAAs can be found that generally present higher expression in cancer cells. However, they are, more often than not, also coexpressed in low levels in non-malignant tissues, enabling dangerous cross-reactivity and on-target off-tumor toxicity [75,79,80]. This also means that CAR T cell therapies that end up proceeding to clinical trials, although deemed safe enough in preclinical testing, often spur cases of severe and sometimes deadly toxicity, leading to product failure [76,81]. Even when dealing with TAAs of very low expression in healthy cells, solid tumors are usually very heterogeneous, so wide antigen expression variability and antigen-loss events are quite common [80].

Should these hurdles be overcome, the outlook for remission and longer-term survival rates in virtually all types of advanced cancer patients will gain a unique chance to ameliorate.

### 6.2. Safety

The success of adoptive CAR T cell transfer is undeniable. However, treatment responses, for now, come at a cost of a high risk of severe, life-threatening early-on adverse effects, be they systemic cytokine storm-related events or wrongful targeting of healthy tissues. These adverse reactions originate primarily from the overactivation and overstimulation of CAR T cells and other involved immunological players—in the case of CRS and neurotoxicity, or from on-target off-tumor toxicity caused by CAR T cell cross-reactivity provoked by cognate antigen coexpression in normal cells. Clinicians often have to resort to therapeutic interventions to attenuate these side effects, while trying to preserve the function and efficacy of the administered CAR T cells [82].

Capability of prevention, as opposed to remediation, of these serious and sometimes fatal adverse reactions would reinvigorate the output of CAR T cell therapies to the market.

#### 6.2.1. Cytokine-Release Syndrome

CRS is a type of cytokine storm syndrome, graded between I and IV depending on symptomatic severity, caused by exaggerated levels of circulating inflammatory cytokines, such as IL-6 and IFN-γ. Mild cases often present flu-like, systemic inflammatory response symptoms, such as fever, fatigue, and generalized pain. Severe cases, however, are characterized by “hypotension as well as high fever and can progress to an uncontrolled systemic inflammatory response with vasopressor-requiring circulatory shock, vascular leakage, disseminated intravascular coagulation, and multi-organ system failure” [83], which require urgent medical attention and sometimes culminate in patient death. Clinical trials of anti-CD19 CAR T cells in blood cancers have often reported high frequencies of CRS, sometimes as high as 100%, and related fatalities. An apparent connection between tumor burden and severity of CRS reactions has been reported several times regarding CAR T cell treatments [83,84]. Haemophagocytic lymphohistiocytosis (HLH) and macrophage activation syndrome (MAS) are even more severe adverse reactions also associated with CAR T cell therapy, which present similar symptoms to CRS, but with added “elevated serum levels of ferritin and liver enzymes, haemophagocytosis, cytopenias, renal failure, pulmonary oedema, splenomegaly and/or an absence of NK cell activity” [25].

Tocilizumab (RoActemra), an anti-IL-6 receptor monoclonal antibody, is the most commonly utilized treatment for CRS, followed by other antibody-based strategies and corticosteroids. However, not all CRS events are responsive to this therapy and HLH/MAS can be especially refractory to it [25,82,84].

#### 6.2.2. Neurotoxicity

Adverse CAR T cell-induced neurotoxicity is nowadays referred to as immune effector cell-associated neurotoxicity (ICANS), and it is reported to occur in approximately two-thirds of leukemia and lymphoma patients treated with adoptive CAR T cell transfer. Although its pathophysiology is still uncertain, general clinical understanding states that exacerbated immune activation and elevated serum and cerebrospinal fluid cytokines (also related to CRS) play an important role in blood–brain barrier dysfunction and neurotoxicity [25,85].

ICANS can present itself clinically through “expressive aphasia, tremor, dysgraphia, and lethargy; these symptoms can progress to global aphasia, seizures, obtundation, stupor, and coma” [86] and, as can be expected, often follows or occurs concomitantly with events of CRS. Administration of corticosteroids, like dexamethasone, is the preferred mitigative ICANS treatment, but there are conflicting reports on the benefits or harmfulness of tocilizumab therapy for this condition [25,85,86].

#### 6.2.3. On-Target Off-Tumor Toxicity

On-target off-tumor toxicity occurs when the cognate antigen of the CAR T cells is expressed not only in the tumoral target, but also in normal cells. An example of this effect is the B cell aplasia commonly caused by commercially available CAR T cell products.

As the potent, genetically modified T lymphocytes disseminate through the blood and infiltrate the various tissues of the body, they encounter all types of cells:

- normal ones, with no expression of the specific antigen, that go by immunologically invisible.
- target malignant cells, which highly express the antigen and are therefore attacked by the lymphocytes.
- problematic, target antigen-negative malignant cells, which also often escape unscathed from the cytolytic action of CAR T cells.
- normal cells expressing the target antigen, which, unfortunately, get caught in the immune crossfire and end up succumbing to the inflammatory and cytolytic action of the CAR T cells—this “friendly-fire” can have serious consequences, damaging healthy tissue and compromising its function, besides creating unnecessary inflammation, which can have detrimental effects both locally and systemically [87].

This premise limits CAR constructs in terms of target recognition. With the lack of a perfect, tumor-circumscribed TAA, standard CARs should only possess low-avidity LBDs or target a TAA which is only co-expressed in nonessential tissues (e.g., the thymic stroma) [87,88].

#### 6.2.4. Other Safety Concerns

Other types of safety concerns that exist regarding CAR T cell therapies which practitioners must be on the lookout for in the days following CAR T cell infusion include anaphylaxis, graft-versus-host disease (when considering possible allogeneic treatments), unpredictable off-target toxicity, and, to a lesser extent, hypothetical viral insertional oncogenesis (when viral vectors are used) [89].

## 7. Innovative Research

Confronting the many challenges still faced by adoptive CAR T cell therapies, exciting projects are leading innovation efforts to improve efficacy, safety, and applicability. Several distinct approaches attempt to resolve various problematic aspects of CAR T treatments. Some include strategies to shorten the lengthy manufacturing period for autologous infusion [90]; others use low-affinity CARs that reduce on-target off-tumor toxicity [91]; others target tumor-supporting cells like Tregs, TAMs, and MDSCs [92]; and others potentiate therapy by adding stimulators like bispecific T cell engagers (BiTEs), marked antibodies [25], etc. This chapter, however, will focus on yet another promising set of concepts of impressive ingenuity.

As mentioned previously, nomenclature in this recent field is yet to be fully agreed upon. Thus, some of the nomenclature utilized hereinafter (noted whenever necessary) does not stem from scientific consensus but from personal preferences and interpretations of the underlying concepts.

**Figure 4:**
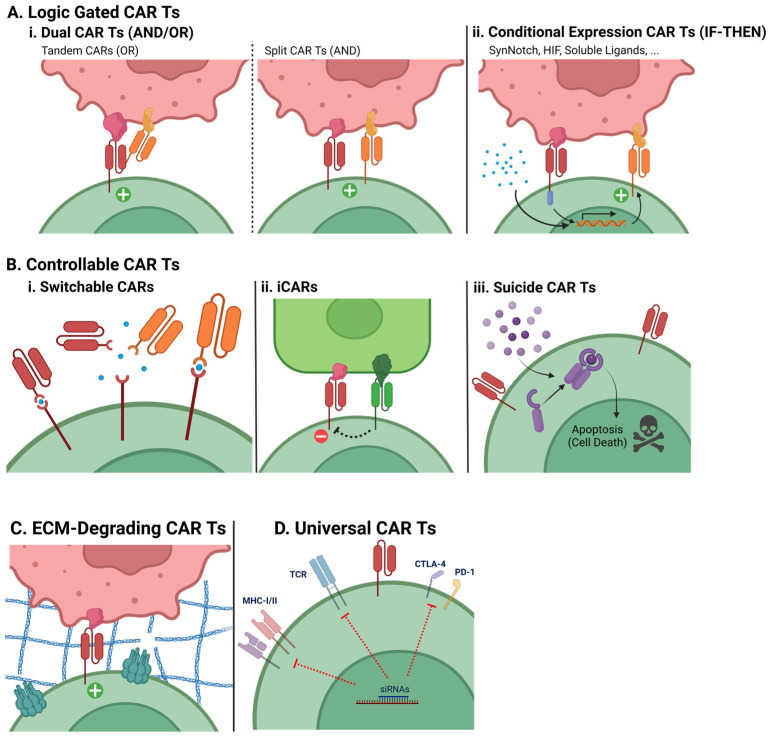
Visual representation of special CAR T engineering designs. Receptor endodomain regions are represented simplistically as a single straight line in the cytoplasmic part of the depicted membrane proteins. The plus (+) sign signifies full CAR T activation signaling, and the minus (−) sign signifies inhibition of CAR activation signaling.

Clarifying schematic representations of the explored CAR T engineering strategies are provided in Figure 4.

### 7.1. Special CAR T Engineering

#### 7.1.1. Logic-Gated Strategies

Logic-gated CAR T cells activate according to logical (or boolean) operators, through unusual CAR signaling strategies or modifications to the expression system. While AND and IF-THEN operator strategies help to reduce off-tumor toxicity, OR operator strategies enhance efficacy by broadening the target cell pool.

**Dual CAR T cells (AND/OR)**

Dual CAR T cells possess two distinct LBDs, which can be linked in the same CAR or coexpressed from different CARs altogether. At least three strategies fit into this category.

The first strategy is called a tandem CAR, consisting of a single CAR protein that exposes two linked LBDs, allowing the recognition of either cognate antigens to activate the CAR T (OR operator). This means that the CAR T cells can target two or more distinct cell populations simultaneously. Another strategy is simply a CAR T cell expressing two independent CAR constructs (another OR operator). Thirdly, the expression of two “co-dependent” CARs in a single split–dual CAR T only allows activation when both cognate antigens are recognized simultaneously. The latter is made possible by the separation (splitting) of the CD3ζ activating domain and the costimulatory domains between the two CARs—it is only when both CARs are stimulated that both signals 1 and 2 fully activate the lymphocyte. This is particularly useful in cases where one of the TAAs has considerable expression in healthy tissues, because split CAR T cells will not be able to target the healthy cells based on that antigen’s expression alone: a secondary antigen more specific to the malignant cells will restrict the CAR T cells’ activity and direct them only to the tumor cells [93].

**Conditional Expression CAR T cells (IF-THEN)**

Conditional expression CAR T cells only express the usual CAR construct after exposure to a given stimulus. This stimulus can be a soluble ligand; however, it is viable to make these cells express two distinct CARs: a “priming” CAR lacking CD3ζ and instead capable of inducing expression of a second, “true” CAR. This last type of conditional expression CAR is also considered to function as a particular type of AND operator, since it requires the presence of two antigens. However, it is the recognition of the first antigen by the priming CAR that induces expression of the true CAR construct, which may then recognize the second antigen and activate the CAR T cell. Thus, if the first antigen is recognized, then the usual CAR T cell function is enabled. Some pathways utilized as CAR T cell primers are SynNotch and HIF [88,94].

The result of this strategy is that CAR T cells will behave in a similar fashion to the split CAR T cells, sparing any non-malignant cells.

#### 7.1.2. Controllable CAR T cells

(Personal nomenclature, unofficial)

This therapeutic cell set consists of CAR T cells that possess mechanisms through which either practitioners or non-malignant cells’ expression can modulate their action.

**Switchable CARs**

(Non-consensual nomenclature. Sometimes called “Universal CARs” or “Split CARs” in the literature, which is misleading due to the existence of other universal CARs and split CARs, also covered in this section)

As their name attempts to imply, these CARs can have a module swapped out for another, namely the LBD. This is accomplished by creating a CAR which, instead of a standard LBD, has an ectodomain that binds to a modified soluble LBD through a small ligand. This allows for the CARs’ targeting to be altered by infusing different LBDs into the patient and controlling the small ligand’s concentration. This strategy is particularly useful since it allows for the redirection of CAR T cell targeting towards subpopulations of cancer cells that escape the first targeting, reducing the appearance of antigen-escape phenomena [76,95,96].

**Inhibitory CARs**

iCAR constructs serve as an accessory to the main CAR. Briefly, whenever an iCAR binds to its target, it activates inhibitory pathways (much like physiological inhibitors, such as CTLA-4), blocking any activation signals stemming from the main CAR. In essence, CAR T cells possessing iCARs will not be able to activate in the presence of healthy cells coexpressing the main CAR’s cognate antigen and the inhibitory antigen (an antigen that, ideally, only malignant cells do not express). Once again, this type of strategy reduces off-tumor toxicity [97].

**Suicide CAR T cells**

As can be inferred from their designation, suicide CAR T cells have an embedded “off-switch”. For example, they can express a ligand-inducible caspase that dimerizes and activates when in said ligand’s presence, initiating T cell apoptosis. By utilizing this strategy, practitioners can safely and accurately shut down unwanted or exaggerated CAR T cell response on demand just by administering the patient with the appropriate ligand [26,98].

#### 7.1.3. ECM-Degrading CAR T Cells

Previously, regarding the unsuccess of CAR T cell therapies against solid tumors, the role of the tumor stroma and associated ECM was mentioned. Nowadays, novel and exciting strategies can provide CAR T cells with the necessary tools to deal with the blockades posed by the tumor microenvironment. One of these strategies is the genetic modification of the lymphocytes to express not only the CAR but also ECM-degrading enzymes, such as heparanase (HPSE). In fact, research shows that HPSE^+^ CAR T cells maintain their existence and expansion, while having an added ECM-degrading functionality that imparts to them greater antitumor capacity against ECM-rich targets [99].

Should ECM-degrading CAR T cell products find their way into the market, treatment efficacy for advanced solid cancer patients could rise considerably and positively affect overall survival.

**Universal CAR T cells**

(Non-consensual nomenclature)

The rising need for quick and mass manufacturing of CAR T cells, due to rapidly growing applicability, has shone light on problems regarding the use of autologous CAR T cells. Expansion of CAR T cells is slow and can be troublesome because of lymphocytes’ poor condition or low count due to previous chemotherapy and lymphodepleting regimens, and it drives up therapy costs to amounts that can be too steep for patients to afford [100]. Attempts at developing “off-the-shelf”, allogeneic products are therefore increasing, with immunogenicity and GVHD reigning as priority concerns. Universal CAR T cells, or UCARTs, are allogeneic CAR T cells engineered to have reduced or no expression of highly immunogenic markers and GVHD-inducing proteins. This can be achieved, for example, using siRNAs to silence the expression of MHC-I, MHC-II, TCR, and immune checkpoint molecules in the donor cells. This silencing renders them less visible to the host’s immune system, preserving the product’s efficacy, while protecting the host from the immunological response naturally hard-wired into the donor cells. TALEN gene editing can also be employed for this purpose [28,101,102].

### 7.2. CAR T Cell Therapy beyond Cancer

Although this review focuses solely on the impact of CAR T cell therapy in the field of cancer treatment, new applications are being investigated for this useful tool. For example, in the treatment of refractory systemic lupus erythematosus, anti-CD19 CAR T cells can be infused into patients to induce effective B cell aplasia and hypogammaglobulinemia, which, as previously noted, is typical of these products. Research has highlighted that, after CAR T infusion, remission was observed and maintained even after normal B cell reappearance [103].

## 8. Conclusions

Chimeric antigen receptor T cells, a phenomenal feat of bioengineering ingenuity, have brought about incredible treatment opportunities and refinement to the fields of medical oncology and more. Several advanced-stage leukemia and lymphoma patients now get the chance to have an increasingly more optimistic prognosis, even when standard treatment has, unfortunately, failed their needs. Solid malignancies will be sure to follow as knowledge accumulates and technology develops. While unresolved issues still block the full potential and applicability of CAR T cells (mainly surrounding CAR T refractory disease and therapy safety), the numerous modifications and enhancements that can be engineered into constructs and the elegant strategies that accompany them allow for a constant evolution of cutting-edge research, which will undoubtedly continue to take these therapies one step further towards new ground and amazing breakthroughs.

## Figures and Tables

**Table 1:** List of the available CAR T cell products, available in either Europe, under the EMA, or the USA, under the FDA (abbreviations in footer).

Commercial Name	Product Name	Manufacturer	Application	Approval
Yescarta	Axicabtagene ciloleucel (anti-CD19)	Kite Pharma, Inc. (Los Angeles, CA, USA)	LBCL	EMA and FDA
			HGBCL	FDA
			PMBCL	EMA and FDA
			FL	EMA and FDA
Kymriah	Tisagenlecleucel (anti-CD19)	Novartis Pharmaceutical Corporation (Basel, Switzerland)	LBCL	EMA and FDA
			HGBCL	FDA
			FL	EMA and FDA
			B-ALL	EMA and FDA
Breyanzi	Lisocabtagene maraleucel (anti-CD19)	Juno Therapeutics, Inc. (Bristol-Meyers Squibb) (Seattle, WA, USA)	LBCL	EMA and FDA
			HGBCL	FDA
			PMBCL	EMA and FDA
			FL3B	EMA and FDA
Tecartus	Brexucabtagene autoleucel (anti-CD19)	Kite Pharma, Inc. (Los Angeles, CA, USA)	MCL	EMA and FDA
			B-ALL	FDA
Abecma	Idecabtagene vicleucel (anti-BCMA)	Celgene Corporation (Bristol-Meyers Squibb) (Summit, NJ, USA)	MM	EMA and FDA
Carvykti	Ciltacabtagene autoleucel (anti-BCMA)	Janssen Biotech, Inc. (Beerse, Belgium)	MM	EMA and FDA

CD19—Cluster of differentiation 19; LBCL—Large B cell lymphoma; EMA—European Medicines Agency; FDA—Food and Drug Administration; HGBCL—High-grade B cell lymphoma; PMBCL—Primary mediastinal large B cell lymphoma; FL(3B)—Follicular lymphoma (Grade 3B); B-ALL—B cell acute lymphocytic leukemia; MCL—Mantle cell lymphoma; BCMA—B cell maturation antigen; MM—Multiple myeloma. Information retrieved from EMA’s (https://www.ema.europa.eu/en (accessed on 28 October 2022)) and FDA’s (https://www.fda.gov/ (accessed on 28 October 2022)) official websites.

**Table 2:** Overview of the clinical results of currently available CAR T cell products and some concepts undergoing clinical trials.

CAR T	Cancer Type	Results	Status
CD19-targeting CARs (Yescarta, Kymriah, Breyanzi, Tecartus)	Various lymphomas and leukemias	**Yescarta:** overall survival at 12 months of 65%, 42.6% at 5 years [37,38]. **Kymriah:** 82% remission rate, 5-year relapse-free survival rate of 44% and 5-year overall survival rate of 55% [39]. **Breyanzi**: event- and progression-free survival of 10.1 months and 14.8 months, respectively (NCT03575351). **Tecartus**: overall survival of 25.5 months and remission rate of 85% at 24 weeks [40].	Commercially Available Some PI/IICTs: (NCT04227015) (NCT04844086) (NCT05470777)
BCMA-targeting CARs (Abecma, Carvykti)	Multiple myeloma	**Abecma**: overall and progression-free survival of 12.5 months and 8.5 months, respectively. A total of 82% of patients developed some grade of CRS, but only 3% had it at grade 3 or higher [41]. **Carvykti**: very good partial responses or complete responses were achieved in 66.7% of patients in a PIICT (NCT4133636). After 13.5 months, only one patient retained a clinical response. A total of 83.3% of the patients presented with some grade of CRS, up to grade 4 for one patient.	Commercially Available Some PI/IICTs: (NCT03767751) (NCT03448978) (NCT03361748)
CD20-targeting CARs	Various lymphomas and leukemias	Six complete remissions, three partial remissions and two stable diseases in 11 patients in a PIICT (NCT01735604), with a median progression-free survival of 6 months.	Some PI/IICTs: (NCT04007029) (NCT04697940)
CD22-targeting CARs	Various lymphomas and leukemias	In a PICT (NCT04088890), three patients (100%) with recurrent malignancies after CD19-targeting CAR T cell therapy achieved complete remission. Adverse events such as grade 1 and 2 CRS and high-grade neutropenia, thrombocytopenia, and anemia were detected.	Some PI/IICTs: (NCT05470777) (NCT05507827)
IL13Rα2-targeting CARs	Glioblastoma	A 228-day-long regression in one patient (PICT NCT02208362). Recurrence of cancer at four new locations, probably due to reduced TAA expression [42].	Some PICTs: (NCT04003649) (NCT04661384) (NCT02208362)
Allogeneic NKG2D-based CAR (CYAD-101)	Metastatic colorectal cancer	In a PICT (NCT03692429) of 15 patients, 2 partial responses and 9 stable diseases were achieved, 7 of which lasted at least 3 months. Median progression-free survival: 3.9 months [43].	Some PICTs: (NCT03692429) (NCT04991948)
HER2-targeting CARs	HER2+ cancers (pancreatic, breast, gastric, others)	In an advanced pancreatic cancer PICT (NCT01935843) of 11 patients, a 4.5 month-long partial response and 5 stable diseases were achieved. Median progression-free survival: 4.8 months (range, 1.5–8.3 months) [44].	Some PI/IICTs: (NCT04650451) (NCT04430595) (NCT04650451)
GPC3-targeting CARs	Hepatocellular carcinoma	In two advanced hepatocellular carcinoma PICTs (NCT02395250 and NCT03146234), of a total of 13 patients, 2 partial responses were obtained. One patient with stable disease was alive after 44.2 months. Overall survival: 50.3% (6 months), 10.5% (3 years). One grade 5 and several grade 1/2 CRS events were recorded [45].	Some PI/IICTs: (NCT03198546)

For commercially available therapies, some results displayed are representative cases and earlier clinical trials, due to limited real-world data assessments. CRS—Cytokine-release syndrome; PICT—Phase I clinical trial; PIICT—Phase II clinical trial. Information on clinical trials retrieved from www.clinicaltrials.gov (accessed on 10 March 2023).

## Data Availability

No new data were created or analyzed in this study. Data sharing is not applicable to this article.
